# Toxicity of anticancer drugs in human placental tissue explants and trophoblast cell lines

**DOI:** 10.1007/s00204-020-02925-w

**Published:** 2020-10-20

**Authors:** Gaby A. M. Eliesen, Hedwig van Hove, Maartje H. Meijer, Petra H. H. van den Broek, Jeanne Pertijs, Nel Roeleveld, Joris van Drongelen, Frans G. M. Russel, Rick Greupink

**Affiliations:** 1 Department of Pharmacology and Toxicology, Radboud Institute for Molecular Life Sciences, Radboud University Medical Center, (Route 137), PO Box 9101, 6500 HB Nijmegen, The Netherlands; 2 Department for Health Evidence, Radboud Institute for Health Sciences, Nijmegen, The Netherlands; 3 Department of Obstetrics and Gynecology, Radboud Institute for Health Sciences, Radboud University Medical Center, Nijmegen, The Netherlands

**Keywords:** Placental toxicity, Cytostatic drugs, Tyrosine kinase inhibitors, Placental tissue explants, Trophoblasts, Pregnancy

## Abstract

The application of anticancer drugs during pregnancy is associated with placenta-related adverse pregnancy outcomes. Therefore, it is important to study placental toxicity of anticancer drugs. The aim of this study was to compare effects on viability and steroidogenesis in placental tissue explants and trophoblast cell lines. Third trimester placental tissue explants were exposed for 72 h (culture day 4–7) to a concentration range of doxorubicin, paclitaxel, cisplatin, carboplatin, crizotinib, gefitinib, imatinib, or sunitinib. JEG-3, undifferentiated BeWo, and syncytialised BeWo cells were exposed for 48 h to the same drugs and concentrations. After exposure, tissue and cell viability were assessed and progesterone and estrone levels were quantified in culture medium. Apart from paclitaxel, all compounds affected both cell and tissue viability at clinically relevant concentrations. Paclitaxel affected explant viability moderately, while it reduced cell viability by 50% or more in all cell lines, at 3–10 nM. Doxorubicin (1 µM) reduced viability in explants to 83 ± 7% of control values, whereas it fully inhibited viability in all cell types. Interference with steroid release in explants was difficult to study due to large variability in measurements, but syncytialised BeWo cells proved suitable for this purpose. We found that 1 µM sunitinib reduced progesterone release to 76 ± 6% of control values, without affecting cell viability. While we observed differences between the models for paclitaxel and doxorubicin, most anticancer drugs affected viability significantly in both placental explants and trophoblast cell lines. Taken together, the placenta should be recognized as a potential target organ for toxicity of anticancer drugs.

**Electronic supplementary material:**

The online version of this article (10.1007/s00204-020-02925-w) contains supplementary material, which is available to authorized users.

## Introduction

Obtaining safety data on the use of drugs during pregnancy is difficult for several reasons. First, pregnant women are generally excluded from clinical drug efficacy and safety studies for ethical reasons. Hence, when a drug enters the market, the only safety data available stem from mandatory animal reproductive toxicity studies, since non-animal models of pregnancy are currently not incorporated in standard preclinical toxicity testing. With regard to the placenta, animal models have poor translational value, as placentation is highly diverse among mammals. For example, the human placenta has a villous structure with a single syncytiotrophoblast layer and an extensive endometrial invasion of extravillous trophoblasts. These features are not present in placentas of commonly used laboratory animals, such as mice or rats (Carter 2007), illustrating the need for human-based preclinical models.

The placenta is essential in maintaining a healthy pregnancy. It fulfills various important functions among which the exchange of nutrients between the mother and fetus and the synthesis of steroids and polypeptide hormones. Also, the placenta expresses a wide variety of xenobiotic transporters and acts as a barrier in protecting the fetus from harmful substances. Impaired placental development and function have been associated both with maternal pregnancy complications, such as preeclampsia (Brosens et al. 2011) and gestational diabetes (Gabbay-Benziv and Baschat 2015), and with adverse pregnancy outcomes, such as prematurity, low birth weight (Brosens et al. 2011; Higgins et al. 2015), and specific birth defects, such as hypospadias (van der Zanden et al. 2012). The placenta is an important site of drug transport because its vascularization is developed for the efficient exchange of nutrients, respiratory gases and waste products. As a result of drug exposure, interference with placental function or development may occur and indirectly affect fetal development (Buerki-Thurnherr et al. 2018; Gohner et al. 2014; Myllynen et al. 2005). Hence, placental toxicity of drugs is an important parameter to study with respect to drug safety in pregnancy.

When cancer coincides with pregnancy, pharmacological treatment is generally favored over pregnancy termination or elective preterm delivery (Maggen et al. 2020). The use of chemotherapy in the first trimester of pregnancy is usually avoided, due to increased risks of miscarriages and congenital malformations (National Toxicology Program 2013). When chemotherapy is applied in the second or third trimester, however, the risk of congenital malformations is equal to the risk in the general population (de Haan et al. 2018; Maggen et al. 2020). Treatment with classical chemotherapeutics, in particular doxorubicin, paclitaxel, cisplatin and carboplatin in single or combination regimens is considered relatively safe from 15 weeks of gestation onwards (Peccatori et al. 2013). Despite their important role in cancer treatment, the use of tyrosine kinase inhibitors during pregnancy is still rare and human safety data on these compounds are limited (Lambertini et al. 2015). An exception is imatinib, which is being used for treatment of pregnant women with chronic myeloid leukemia (Pye et al. 2008). The increased use of chemotherapy over the past 20 years seems to have resulted in a higher proportion of live births (99% of 1170 pregnancies) (de Haan et al. 2018). However, in the same study, the reported proportion of neonates that were small for gestational age (SGA) was 21% and associated with chemotherapy treatment, in particular platinum-based exposure (de Haan et al. 2018). SGA pregnancies are related to impaired placental angiogenesis (Kwiatkowski et al. 2018) and other forms of placental insufficiency, such as diminished nutrient transfer to the fetus (Maulik et al. 2006).

Given the increased use of anticancer treatment during pregnancy and the potential risk of placenta-related adverse pregnancy outcomes, it is increasingly important to investigate the placental toxicity of anticancer drugs. This may eventually be of use in choosing the most appropriate treatment regimen. Anticancer drugs may cause toxic effects on the placenta given their mode of action. The placenta is a highly proliferative organ and trophoblast cells are capable of growing into the endometrium invasively similar to tumor cells. Furthermore, placental trophoblast cells share numerous molecular pathways with tumor cells, and both cell types are known to highly express a variety of tyrosine kinase receptors (Ferretti et al. 2007). Therefore, the placenta may be prone to toxic effects exerted by classical cytostatic drugs targeting rapidly dividing and proliferating cells, but also by more novel tyrosine kinase inhibitors (TKIs) acting specifically on overexpressed tyrosine kinases in tumor cells.

The placenta is a unique organ for human studies because it is readily available after delivery. Since the tissue can endure hypoxic conditions, it is suitable for ex vivo culturing (Miller et al. 2005). When culturing placental villous tissue explants, the tissue microstructure containing multiple placental cell types is maintained. Therefore, this primary tissue model closely resembles the physiological situation and has been proven useful for the study of placental metabolism, oxygen consumption, endocrine function, and drug toxicity. We assessed toxicity of anticancer drugs both in placental villous explants and in the more conventionally used choriocarcinoma cell lines BeWo and JEG-3. BeWo cells are regarded as a cytotrophoblast model with the potential to differentiate into syncytiotrophoblasts upon elevation of intracellular cAMP levels (Orendi et al. 2010), while the HLA-G-positive JEG-3 cells are commonly applied in invasion assays because of their extravillous trophoblast characteristics (Lee et al. 2016). More specifically, we aimed to compare the effects of several classical cytostatic drugs as well as tyrosine kinase inhibitors on viability and steroidogenesis in placental tissue explants and choriocarcinoma trophoblast cell lines.

## Methods

### Anticancer drugs and chemicals

Crizotinib, sunitinib malate, cisplatin, carboplatin, doxorubicin and paclitaxel were purchased from Sigma-Aldrich^®^ (St. Louis, MO, USA). Imatinib and gefitinib were purchased from Cayman Chemical (Ann Arbor, MI, USA). Stock solutions for the TKIs, doxorubicin, and paclitaxel were prepared in dimethyl sulfoxide (DMSO) with maximal final concentrations of 1%. The stock solutions for cisplatin and carboplatin were prepared in culture medium, since DMSO interacts with the platinum complexes resulting in inactivation (Hall et al. 2014).

### Placental villous explant culture

Term placentas, obtained from consenting women following uncomplicated pregnancies and delivered vaginally or by caesarean section, were used for all experiments. Approval of the Regional Committee on Research involving Human Subjects was obtained (File 2014-1397). Within 60 min after delivery, 1 cm^3^ villous tissue samples were excised at random areas midway between the umbilical cord and distal edge of the placenta. To remove maternal blood, samples were carefully rinsed in sterile Dulbecco’s phosphate-buffered saline supplemented with calcium chloride and magnesium chloride (Sigma-Aldrich, St. Louis, MO, USA) which was kept at 37 °C. The villous tissue was cut into small pieces weighing approximately 5 mg, after which 4–6 pieces of tissue were cultured together in individual wells of a 6-well plate. The explants were cultured in 1.5 ml of RPMI-1640 culture medium (Gibco, USA) with 15% heat-inactivated fetal bovine serum, 1% penicillin streptomycin, 1% gentamicin, and 1% amphotericin B (Life Technologies, USA). Placental explant cultures were kept for 7 days at 37 °C in a humidified gas mixture of 5% CO_2_ and 95% air. The culture medium was renewed every 24 h.

### Drug treatment of placental tissue explants

**Fig. 1:**
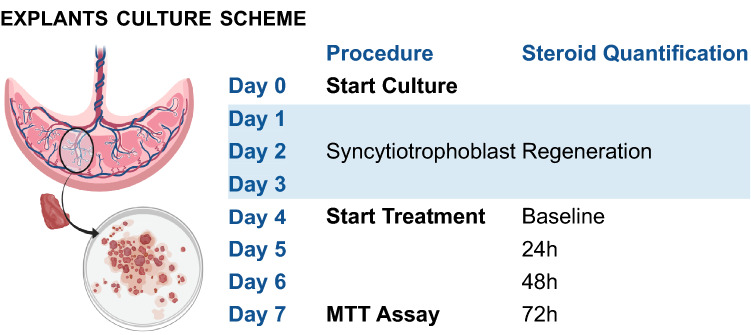
Explants culture scheme. Placental villous tissue was cut into small pieces weighing approximately 5 mg, after which 4–6 pieces of tissue were cultured together in individual wells of a 6-wells plate. Explants were cultured for 7 days and medium was renewed every 24 h. The first 3 days of culture allowed for syncytiotrophoblast regeneration. At day 4, treatment with tyrosine kinase inhibitors or cytostatic drugs was started and medium was stored each day for steroid quantification. At day 7, after 72 h exposure, an MTT assay was performed to measure tissue viability. Created with http://biorender.com/

Treatment with TKIs or cytostatic drugs was started at day four of culture to allow for the syncytiotrophoblasts to regenerate (Siman et al. 2001). Explants were then incubated for 72 h with 1, 10 or 100 µM of all TKIs or cytostatic drugs mentioned above. Cisplatin (1 mM) was used as a positive control (data not shown). For all drugs, apart from cisplatin and carboplatin final DMSO concentrations were 0.1% for the 1 µM and 10 µM conditions and 1% for the 100 µM conditions. For cisplatin and carboplatin, culture medium was used as a solvent. Culture medium was replaced every 24 h with new medium containing the original concentration of the drug. Supernatant was collected each day and stored at − 20 °C for hormone quantification. Figure 1 shows a schematic overview of the experimental procedure.

### MTT assay in placental explants

To evaluate tissue and cell viability, after 72 h of drug exposure, tissue explants were incubated for 4 h at 37 °C with 1 mM 3-(4,5-dimethylthiazol-2-yl)-2,5-ditetrazolium bromide (MTT) in RPMI. After incubation, the medium was removed, the tissue in each well was weighed, and the explants were subsequently placed in 500 µL DMSO for 10 min to dissolve the formazan formed. After 10 min, the DMSO was transferred to a 96-well plate and absorbance was measured at 540 nm using a Biorad Multiwell Plate reader.

### Immunohistochemistry

Placental explants were fixed in 4% formalin in phosphate-buffered saline (PBS). After dehydration, tissue samples were embedded in paraffin and one paraffin block contained approximately four explants. Sections of 4 μM were cut, dried, deparaffinized and rehydrated in ethanol to PBS. These were further processed for standard hematoxylin and eosin staining or immunohistochemical staining to visualize human chorionic gonadotropin (hCG) expression. For immunohistochemistry, antigen retrieval was performed using Tris–HCl buffer at pH 9 at 96 °C for 15 min. Endogenous peroxidase was blocked with 3% H_2_O_2_ in PBS containing 0.1% Tween20 (PBST) for 30 min and aspecific-binding epitopes were blocked with 1% BSA in PBST for 30 min. Sections were incubated overnight at 4 °C with a polyclonal primary antibody detecting hCG (cat. no. A0231, DAKO, Glostrup, Denmark) at a dilution of 10,000×. This was followed by incubation with a secondary swine anti-rabbit polyclonal antibody (cat. no. E0431, DAKO, Glostrup, Demark) for 30 min at room temperature at a dilution of 1000×. Signals were amplified using a standard avidin–biotin complex (Brunschwig Chemie, Amsterdam, The Netherlands) for 30 min at room temperature. Antibody localization was visualized with 3,3′-diamino benzidine and nuclear counterstaining was performed using hematoxylin. Sections were scanned with the Pannoramic 1000 (3DHistech, Budapest, Hungary) and evaluated using the accompanying case viewer software. Microphotographs were made at using the software magnification of 20×.

### Cell culture and syncytialisation

Two human choriocarcinoma-derived placental cell lines were used: JEG3 and BeWo (ATCC catalog numbers HTB-36 and CCL-98, respectively). JEG3 cells were cultured in DMEM and Bewo cells in Ham’s F-12 K medium, supplemented with 10% fetal calf serum and 1% penicillin and streptomycin under a humidified 5% CO_2_/95% air atmosphere at 37 °C. Cells were passaged (JEG3: 1:10 twice a week; BeWo: 1:3 once a week) after trypsinization with 0.05% Trypsin–EDTA when cells reached approximately 80% confluency. For BeWo cells, the medium was renewed 3 times a week. To create syncytialised BeWo cells, regular BeWo cells were treated with 50 μM forskolin (Sigma-Aldrich, St. Louis, MO, USA) in 0.1% DMSO, to initiate syncytialisation. As forskolin increases the intracellular cAMP levels, it activates protein kinase A after which syncytialisation and fusion of trophoblasts occurs (Orendi et al. 2010).

### MTT assay in JEG-3, BeWo, and syncytialised BeWo cells

The cells were seeded in 96-well culture plates at a cell density of 1 × 10^4^ cells/well and cultured for 24 h at 37 °C in a humidified atmosphere with 5% CO_2_ to allow for attachment. Subsequently, the cells were exposed to the TKIs and cytostatic drugs in concentrations ranging from 100 pM to 100 µM (triplicate). For all TKIs, paclitaxel, and doxorubicin, a final DMSO concentration of 0.5% was used. For cisplatin and carboplatin, no DMSO was used in regular BeWO cells and a maximum DMSO concentration of 0.1% DMSO was used in syncytialised BeWo cells. This is a minimum percentage DMSO, but required as cells required co-incubation with forskolin to syncytialise. Crizotinib 100 μM was used as positive control. After attachment, cells were incubated with the compounds for 48 h, after which the medium was removed and the cells were exposed to 1 MTT diluted in medium. After 3 h of incubation with MTT, the formazan crystals formed were solubilized in 50 μL DMSO. The absorbance of formazan was measured at 540 nm by a Biorad Multiwell Plate reader.

### Steroidogenesis and protein assay in syncytialised BeWo cells

For each compound, syncytialised BeWo cells were exposed to three concentrations below the IC50 values with respect to cell viability. For doxorubicin, only two concentrations were tested. Conditioned medium, containing the secreted hormones, was collected after 48 h exposure to the TKIs and cytostatic drugs and stored at − 80 °C until analysis. To eliminate the influence of the varying cell densities between the wells, total protein content in each well was measured. Cells were washed twice with Hank’s buffered Salt Solution (HBSS) and lysed with 50 μL 1 M sodium hydroxide (NaOH). Subsequently, 10 μL of the lysate was mixed with 190 μL of 5 times diluted dye reagent (Biorad protein assay). The absorbance was measured at 595 nm using a Biorad Multiwell Plate reader. The quantities of protein in the samples were determined via a calibration curve of bovine serum albumin.

### Steroid analysis of culture medium

Progesterone and estrone concentrations in culture medium were determined by LC–MS/MS analysis using an Acquity UPLC (Waters, Milford, MA, USA) equipped with a C18 UPLC column (Acquity UPLC BEH C18, 2.1 × 50 mm, Milford, MA, USA, Waters) coupled to a Xevo TQ-S (Waters) triple quadrupole mass spectrometer. Progesterone-D9 was used as internal standard and calibration curves were made in the matrix. To precipitate proteins, methanol containing the internal standard was added to culture medium samples in a ratio 3:1 (MeOH:medium). Following subsequent centrifugation (13,000*g* for 3 min), 1 μl of the clear supernatant was injected into the LC–MS to quantify progesterone and estrone. Solvent A consisted of 0.2 mM NH4F in water and solvent B consisted of 0.2 mM NH4F in Acetonitrile:Methanol in a ratio of 75:25%. Separation was performed at a flow rate of 350 µl/min under the following gradient conditions: 0 min 60% eluent A, 3.0 min 0% eluent A, 4 min 0% eluent A, 5 min 60% eluent A. The effluent from the UPLC was passed directly into the electrospray ion source. Positive electrospray ionization was achieved using nitrogen as a desolation gas with ionization voltage at 600 V. The source temperature was set at 500 °C and argon was used as collision gas. The following SRM transitions were used: *m*/*z* 315.10 (parent ion) to *m*/*z* 69.9 and *m*/*z* 108.9 (both product ions) for progesterone, *m*/*z* 269.0 (parent ion) to *m*/*z* 144.9 and 158.9 (product ions) for estrone, and *m*/*z* 324.2 (parent ion) to *m*/*z* 99.9 and *m*/*z* 112.9 (product ions) for the internal standard d9-progesterone. We also attempted to quantify estradiol and estriol levels in the culture medium of both explants and cells, but these were below the detection limit.

### Data and statistical analyses

**Fig. 2:**
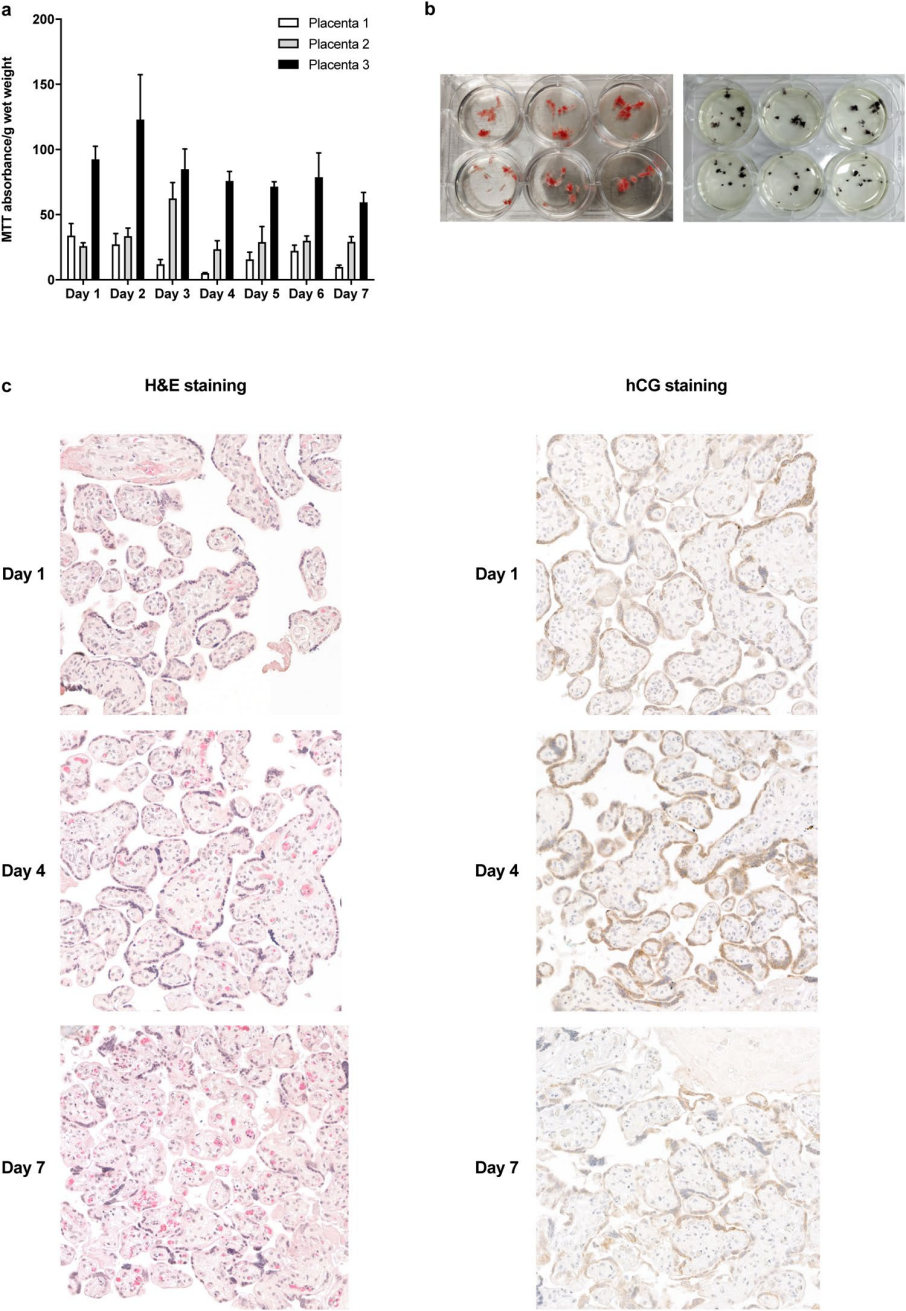
Viability, morphology, and functionality of placental explants over 7 days. MTT absorbance per g wet weight in three individual placentas measured in triplicates (**a**). Bars represent mean ± SD. Explants before (left) and after (right) 3 h of MTT exposure in control conditions (**b**). H&E stainings and hCG immunohistochemical staining of placental tissue on days 1,4 and 7 of culture (**c**). Fetal villi are clearly discernable at all time points but some loss of tissue coherence can be observed at the later time points. Syncytiotrophoblasts stained positive for hCG at all time points

**Fig. 3:**
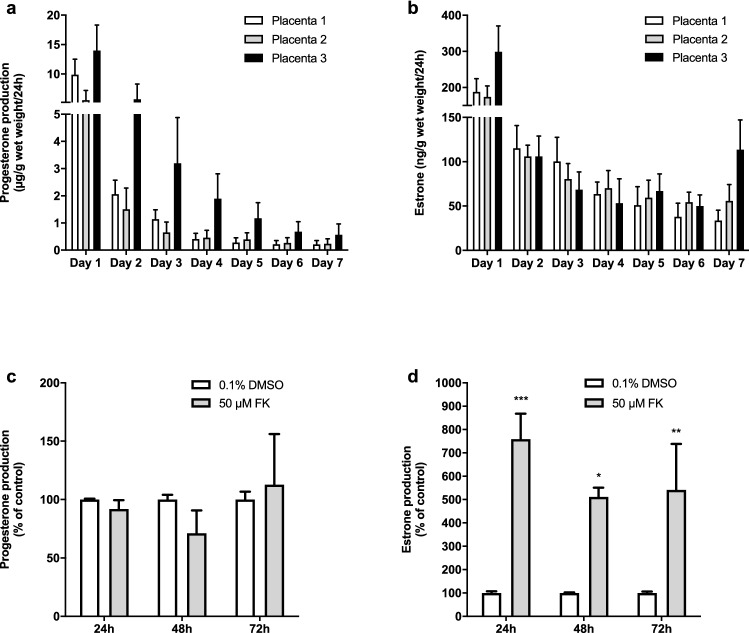
Steroid release in placental explants over 7 days. Progesterone (**a**) and estrone (**b**) release per day in three individual placentas measured in 12 wells. Data points represent mean ± SD (**a**, **b**). Effects of the addition of 50 µM forskolin on relative change in progesterone (**c**) and estrone (**d**) release over 72 h compared to vehicle control (days 5–7). Relative progesterone or estrone release rate per well per 24 h as compared to baseline conditions (0 h) was calculated and normalized against the corresponding control. Bars represent mean + SEM. Statistical analysis was performed with a two-way repeated measures ANOVA and means were compared to control using a Bonferroni post-hoc test. *P < 0.05, **P < 0.01, ***P < 0.001 (**c**, **d**), *FK* Forskolin

Construction of graphs and data analysis were performed using Graphpad Prism version 8.4.2. MTT conversion and progesterone and estrone release in placental explants were normalized to tissue wet weight. To compare the effects on tissue viability and cell viability, the data were normalized to control conditions and mean differences were tested using an unpaired *t* test, a one-way ANOVA, or a two-way repeated measures ANOVA. The change in progesterone release rate as compared to the baseline (at 0 h) was calculated for each well separately, after which data were normalized to control conditions. One-way ANOVA was used to assess effects at the 72 h time point only. The Bonferroni post hoc test was applied to compare treatment conditions with controls. Data are described as mean ± SD in Figs. 2 and 3 and as mean ± SEM in all other figures where comparisons of means between groups were made. The mean and SD or SEM were calculated from all individual results (three experiments performed in duplicate or triplicate wells, *n* = 6 or *n* = 9).

#### Supplementary figures

A repeated measures ANOVA was used to analyze data on steroid release in explants for multiple time points. A regular two-way ANOVA was performed to assess the effect of the presence of DMSO on the inhibitory effects of cisplatin and carboplatin on cell viability. The Bonferroni post hoc test was applied to compare treatment conditions with controls.

## Results

### Tissue viability and steroidogenesis in control conditions in placental explants

The placental explants could be cultured up to 7 days with stable tissue viability and evidence of placental hCG production. The absolute amount of MTT conversion differed between placentas but was stable within a single placenta (Fig. 2a). On the macroscopic level, an apparent purple color was visible as the result of MTT conversion (Fig. 2b). Morphology of the placental explants was mostly preserved over time (Fig. 2c). The presence of villous structures was evident and fetal blood vessels within the villi also preserved their morphology over 7 days. At day 7 of culture, however, some loss of tissue coherence in the villous stroma was observed. In addition, the syncytiotrophoblast layer was less pronounced at the final day of culture and also at day 4, which could be explained by an incomplete regeneration. Still, the positive staining of hCG which confined to the syncytiotrophoblast layer at all time points studied, indicates at least partially maintained integrity and functionality of the syncytiotrophoblast throughout the culture period.

The placental explants released both progesterone and estrone into the culture medium (Fig. 3a and b). Progesterone levels decreased over time from on average 10 ± 4 µg/g wet weight (ww) at day 1 to 0.3 ± 0.2 µg/g ww at day 7. Estrone levels were more stable but 50-fold lower compared to progesterone and were on average 220 ± 70 ng/g ww at day 1 and 70 ± 40 ng/g ww at day 7. Placental explants were responsive to the addition of 50 µM forskolin since a significant induction in estrone release, but not progesterone release, could be observed after 24 h, 48 h, and 72 h exposure (starting at day 4) (Fig. 3c and d).

### Effects of cytostatic drugs and tyrosine kinase inhibitors on tissue viability and steroidogenesis in placental explants

Explants were cultured for 3 days before the start of the treatment to allow for regeneration of the syncytiotrophoblast layer (Siman et al. 2001). Therefore, the fourth day of culture was regarded as the baseline condition (*T* = 0 h) and the seventh day of culture was the final time point (*T* = 72 h, Fig. 1). MTT conversion at baseline did not differ from MTT conversion at 72 h and the addition of 0.1% or 1% DMSO did not affect tissue viability (Fig. S1). No profound or consistent effects of DMSO on progesterone release or estrone release rate could be observed over 72 h.

**Fig. 4:**
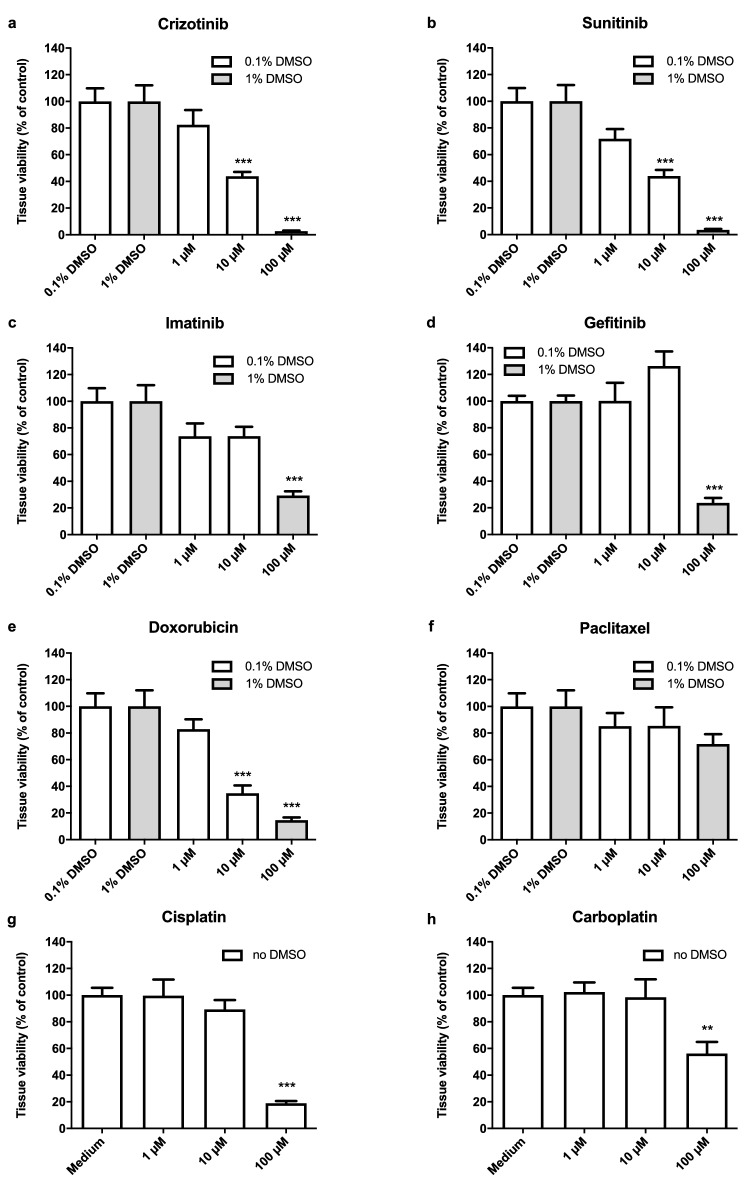
Effects of Tyrosine kinase inhibitors and cytostatic drugs on viability of villous explants. Effects of tyrosine kinase inhibitors (**a**–**d**) and cytostatic drugs (**e**–**h**) on tissue viability. Data were normalized to the corresponding control and bars represent mean ± SEM of three individual experiments each performed in triplicate wells. Statistical analysis was performed with a one-way ANOVA and means were compared to control using a Bonferroni post-hoc test. **P < 0.01, ***P < 0.001

We found that crizotinib, sunitinib and doxorubicin were the most potent inhibitors tested and inhibited tissue viability in a dose-dependent manner after 72 h exposure (Fig. 4). Crizotinib and sunitinib fully inhibited tissue viability, while 100 µM doxorubicin reduced tissue viability to 14 ± 2%. Imatinib, gefitinib, cisplatin, and carboplatin where less potent and reduced tissue viability to 29 ± 3%, 24 ± 4%, 19 ± 2%, and 56 ± 9% of control, respectively, at a concentration of 100 µM. Paclitaxel moderately affected tissue viability, but this was not statistically significant.

**Fig. 5:**
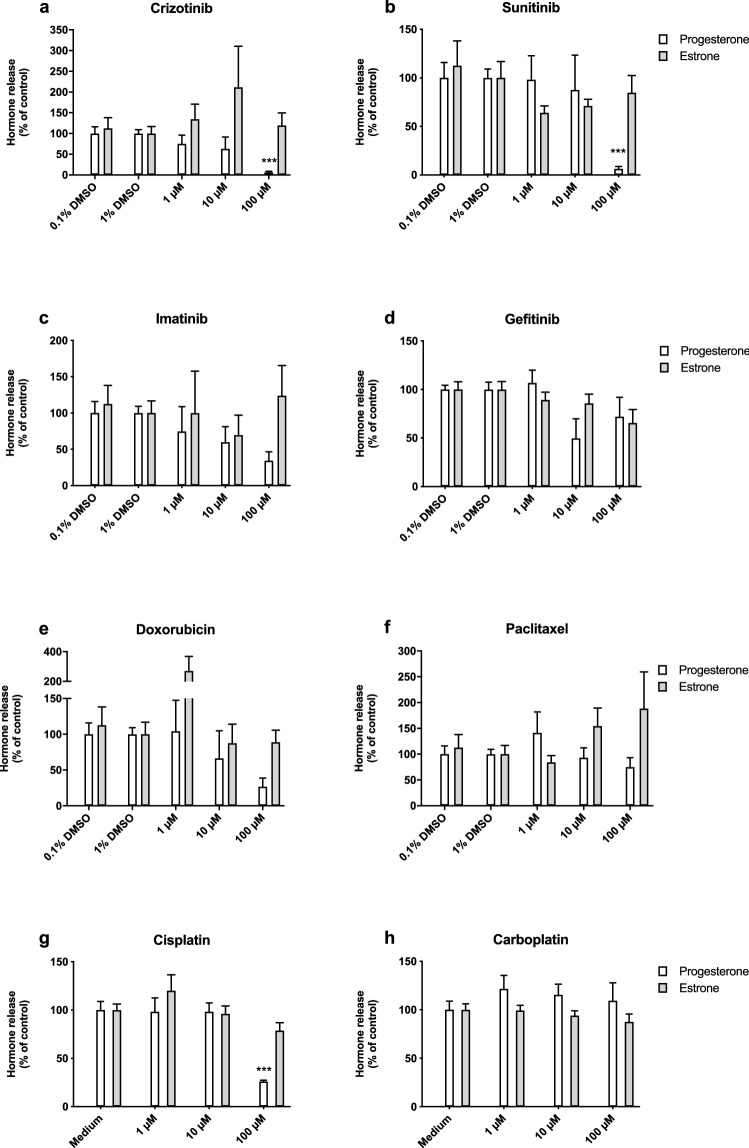
Effects of tyrosine kinase inhibitors and cytostatics on steroid release in villous explants after 72 h exposure. Effect of tyrosine kinase inhibitors (**a**–**d**) and cytostatic drugs (**e**–**h**) on relative progesterone and estrone release rate at 72 h compared to control. Relative progesterone or estrone release rate per well per 24 h (at 72 h) as compared to baseline conditions (0 h) was calculated and normalized against the corresponding control. Bars represent mean ± SEM of three individual experiments each performed in triplicate wells. Statistical analysis was performed per outcome parameter with a one-way ANOVA and means were compared to control using the Bonferroni post-hoc test. ***P < 0.001

Figure 5 displays the estrone and progesterone release in placental explants after 72 h of exposure relative to the pre-exposure time point (0 h) and normalized to the corresponding vehicle control. After 72 h of exposure, a statistically significant reduction in progesterone release was observed after treatment with 100 µM of either crizotinib, sunitinib, or cisplatin (Fig. 5) and this effect was already apparent at earlier time points as well (Fig. S2A). Similar effects were seen for treatment with 10 and 100 µM gefitinib and 100 μM imatinib, but they were only statistically significant if effects over time were analyzed (Fig. S2A). After 24 h, incubation with 1, 10 and 100 µM doxorubicin induced progesterone production (Fig. S2A). For all compounds, a reduction in progesterone release was consistent with a large reduction in cell viability. No substantial effects on estrone release could be observed. At all concentrations applied, including those that were shown to affect cell viability, estrone release remained similar to that in control conditions (Figs. 5, S2B).

### Cell viability and progesterone release in control conditions in JEG-3, BeWo, and syncytialised BeWo cells

**Fig. 6:**
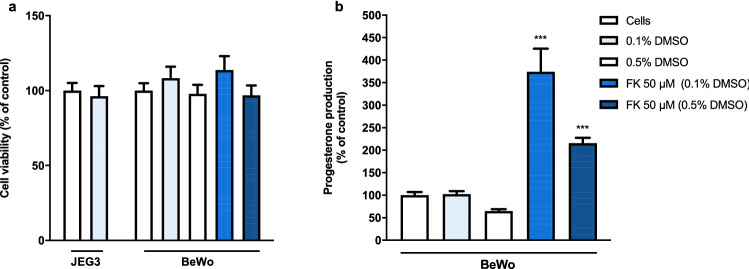
The effects of DMSO and forskolin on cell viability and progesterone release in BeWo cells. DMSO and 50 μM Forskolin (FK) did not alter cell viability in JEG-3 or BeWo cells after 48 h incubation (**a**). Data were normalized against the control (medium, no DMSO) and represent the mean ± SEM of three independent experiments performed in triplicate. The presence of 0.5% DMSO diminished progesterone release in regular and forskolin induced BeWo cells after 48 h incubation (**b**). Data were normalized against the corresponding control and represent the mean ± SEM of three independent experiments performed in triplicate. Mean differences compared to control were assessed using a one-way ANOVA with a Bonferroni post-hoc test. ***P < 0.001

Additionally, we assessed the effect of 48 h treatment with tyrosine kinase inhibitors and cytostatic drugs on cell viability and progesterone release in JEG3, BeWo and syncytialised BeWo. None of the control conditions, nor the addition of forskolin, affected cell viability (Fig. 6a). Furthermore, the presence of 0.1% DMSO did not influence the inhibitory effects of cisplatin and carboplatin on cell viability (Fig. S4). Figure 6b shows that treatment with 0.1% DMSO did not alter baseline progesterone release, but 0.5% DMSO reduced progesterone release to 64 ± 4% compared to cells without DMSO. Treatment with forskolin induced syncytialisation in BeWo cells and consequently enhanced the progesterone production to 370 ± 50% (Forskolin 50 μM in 0.1% DMSO) or 215 ± 12% (Forskolin 50 μM in 0.5% DMSO).

### Effects of tyrosine kinase inhibitors and cytostatic drugs on cell viability and progesterone release in trophoblasts cell lines

**Fig. 7:**
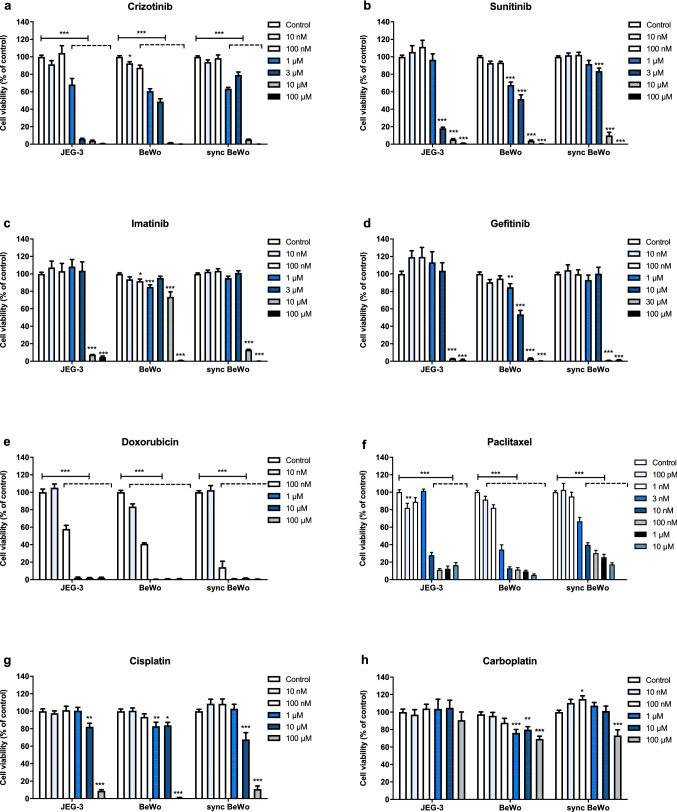
The effects of tyrosine kinase inhibitors and cytostatic drugs on cell viability in JEG3, BeWo and syncytialised BeWo. Effects of tyrosine kinase inhibitors (**a**–**d**) and cytostatic drugs (**e**–**h**) on cell viability in JEG-3, BeWo, and syncytialised BeWo cells after 48 h exposure. Data were normalized to the corresponding control and bars represent mean ± SEM of three individual experiments each performed in triplicate wells. Statistical differences were tested per cell line using a one-way ANOVA and the Bonferroni post-hoc test was used to compare means of the treatment conditions to control. *P < 0.05, **P < 0.01, ***P < 0.001

Apart from carboplatin, all compounds studied inhibited cell viability fully or to less than 20% of control conditions (Fig. 7). While paclitaxel did not affect explant viability, it was the most potent inhibitor in cells and reduced cell viability to 50% or less in all cell types at 3–10 nM. Additionally, it was consistently less cytotoxic in syncytialised BeWo cells as compared to undifferentiated BeWo and JEG3 cells. Doxorubicin was the second most potent inhibitor of cell viability and was approximately 10 times more potent in all cell types than in explants. All TKIs and platinum compounds affected cell and tissue viability to a similar extent. Sunitinib inhibited cell viability at multiple concentrations to a lesser extent in syncytialised BeWo cells as compared to undifferentiated BeWo and JEG3 cells.

**Fig. 8:**
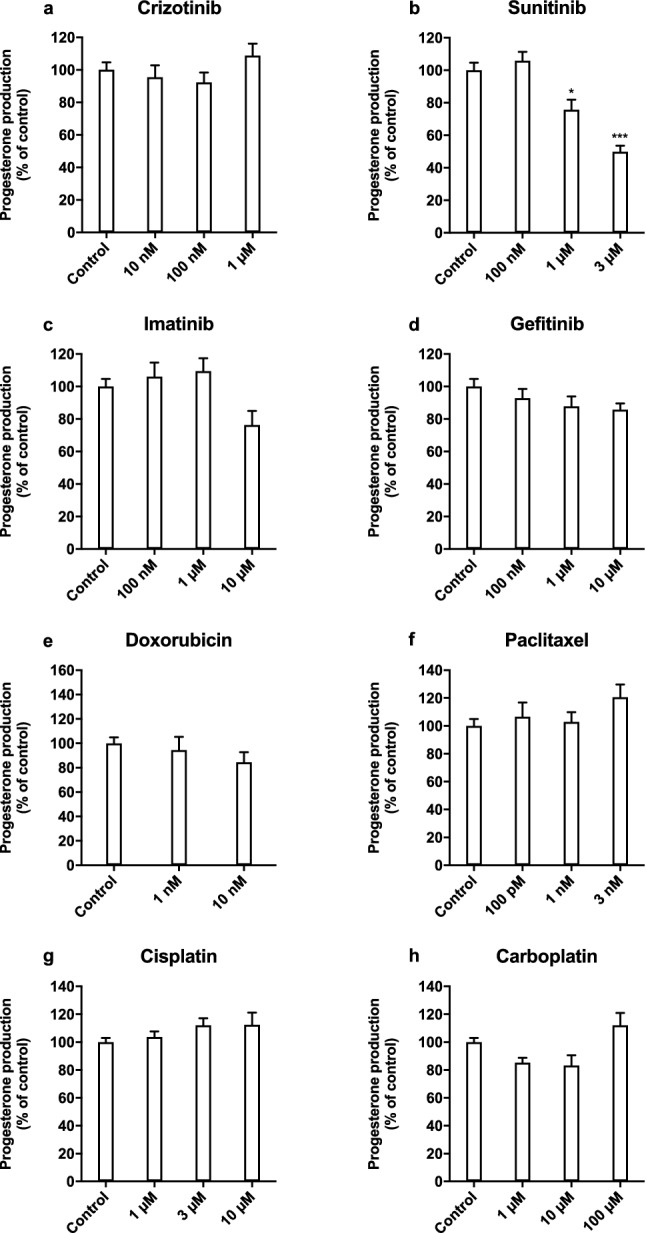
The effects of tyrosine kinase inhibitors and cytostatic drugs on progesterone release in syncytialised BeWo cells. Effects of tyrosine kinase inhibitors (**a**–**d**) and cytostatic drugs (**e**–**h**) on relative progesterone production at 48 h compared to control. Bars represent mean ± SEM of three individual experiments each performed in triplicate wells. Lines represent the corresponding effect on cell viability (Fig. 7) after 48 h exposure. Statistical analysis was performed with a one-way ANOVA and means were compared to control using the Bonferroni post-hoc test *P < 0.05, ***P < 0.001

For each compound, we also assessed the effects on progesterone release in syncytialised BeWo cells using three concentrations below the IC50 value for cell viability (Fig. 8). Sunitinib (1 and 3 µM) reduced progesterone release to 76 ± 6% (*P* < 0.05) and 50 ± 4% (*P* < 0.001) and 10 µM imatinib reduced progesterone release to 76 ± 9% (not statistically significant). Treatment with crizotinib (1 μM), paclitaxel (3 nM), cisplatin (100 μM), and carboplatin (100 μM) resulted in a slight induction of progesterone release, consistent with a reduction in cell viability, but these effects were not statistically significant. The other compounds did not affect progesterone release in the concentrations tested.

## Discussion

We hypothesized that anticancer drugs are potentially toxic to placental tissue due to their mode of action which was indeed true for all compounds studied. All drugs affected tissue viability in explants, with doxorubicin, crizotinib and sunitinib being the most potent inhibitors. The other compounds only started affecting tissue viability when applied at a concentration of 100 μM, while paclitaxel only showed mild, but non-significant effects at this concentration. We then corroborated these findings in three placental trophoblast cell lines. A striking result found was that paclitaxel affected tissue viability in explants only moderately, whereas it proved to be the most potent inhibitor of cell viability in all three cell lines. Similarly, doxorubicin inhibited viability in trophoblast cell lines ten times more potently compared to placental explants. For the platinum-based compounds and all tyrosine kinase inhibitors studied, we did not see any major differences between cell lines and placental explants with respect to inhibitory effects on cell or tissue viability.

The reported effects of anticancer drugs on cell and tissue viability here are of potential clinical relevance in the light of their therapeutic plasma concentrations. For paclitaxel, cisplatin, imatinib, and gefitinib, maximum plasma concentrations after single dose i.v. administration or after multiple oral administrations are between 1 and 10 μM (Di Gion et al. 2011; Scheffler et al. 2011; Sonnichsen and Relling 1994; Urien and Lokiec 2004). Carboplatin concentrations may even be as high as 100 μM (Gaver et al. 1988). The maximum plasma concentrations for crizotinib and sunitinib are between 100 nM and 1 μM (Di Gion et al. 2011; Hamilton et al. 2015) and for doxorubicin between 10 and 100 nM (Speth et al. 1988). As for most of these drugs, plasma protein binding is more than 90%, unbound plasma levels will be considerably lower. To better understand the risks of toxicity, actual drug concentrations in exposed placentas need to be assessed in future studies, since tissue concentrations may deviate from plasma concentrations.

As both doxorubicin and paclitaxel exert their action by interacting with an intracellular target, a lower intracellular concentration in explants may explain the different findings, but we did not quantify tissue or intracellular exposure. Previously, however, paclitaxel and doxorubicin were found to be taken up in ex vivo perfused placental tissue at 3% or 38–70% of initial maternal concentrations, respectively (Berveiller and Mir 2012; Soininen et al. 2015). In in vivo*,* a ten-fold higher paclitaxel concentration was seen in placental tissue compared to maternal levels at delivery (Berveiller et al. 2018) and in vivo placental uptake of doxorubicin has been described in one case (Karp et al. 1983). Since placental exposure is apparent from these studies, a lack of exposure cannot fully explain the observed difference in potency between explants and cell models. Alternatively, the difference may be explained by the mechanism of action through which these drugs acts. Paclitaxel inhibits mitosis by preventing depolymerization of microtubules and doxorubicin is an inhibitor of DNA replication. The cell lines used are tumor cell lines which have a higher proliferation rate than placental tissue explants, allowing toxicities associated with mitotic activity to be picked up more readily. Also, placental mesenchyme or other placental cell types present in explant samples could be less prone to toxicity exerted by classical cytostatic drugs as compared to trophoblasts. Lastly, paclitaxel is a classical substrate of the efflux transporter P-glycoprotein (P-gp) and doxorubicin is a substrate of P-gp, Multidrug-Resistance Protein (MRP) 1 and Breast Cancer-Resistance Protein (BCRP) which could reduce their intracellular exposure. Chronic in vivo exposure to paclitaxel may even increase protein expression of P-gp and BCRP in the placenta (Berveiller et al. 2018). Differences in absolute amounts of these transporters or drug metabolizing enzymes between cell lines and primary cells or tissue explants could explain differences in exposure and effects, but this remains to be elucidated.

Next to studying differences between explants and cells, it is, therefore, also relevant to examine potential differences in observed drug toxicity between the cell types. JEG3 and BeWo cells are commonly used as a model for extravillous trophoblasts and cytotrophoblasts, respectively, or for syncytiotrophoblasts after treatment with forskolin (Lee et al. 2016; Orendi et al. 2010). It should be noted, however, that the gene expression signatures of BeWo and JEG3 cells are quite similar but largely different from primary cytotrophoblasts or extravillous trophoblasts (Bilban et al. 2010). With respect to inhibiting cell viability, we found that both paclitaxel and sunitinib were less potent in syncytialised BeWo cells compared to BeWo and JEG-3 cells. This could imply that syncytiotrophoblasts are slightly less prone to the toxic effects of these compounds on cell viability. This hypothesis is supported by a previous study in which syncytialised BeWo cells were more resistant to cisplatin-induced apoptosis compared to undifferentiated BeWo cells. (Wei et al. 2012). An increased resistance to toxicity was observed only for some of the anticancer drugs studied here and we did not see such an effect for cisplatin. Further studies should elucidate the underlying mechanisms in more detail.

With respect to inhibitory effects on cell viability in general, our findings are in line with literature. For paclitaxel, a 50% reduction in cell viability of isolated third trimester primary trophoblasts was reported with paclitaxel concentrations of 18–180 nM (Berveiller et al. 2018), which is comparable to what we found in syncytialised BeWo cells. In undifferentiated BeWo cells, we saw a 50% reduction in cell viability at 1–3 nM, similar to what has previously been reported (Marth et al. 1995). Previous studies also showed that doxorubicin was taken up by BeWo cells and inhibited cell viability with an IC50 of 0.6 µM after 4 h of exposure (Soininen et al. 2015), in line with our observation after 48 h. Previously, we studied the effects of multiple TKIs on cell viability of BeWo cells and the IC50 values found corresponded with the results we observed here (Eliesen et al. 2017).

The extent to which placental toxicity attributes to adverse pregnancy outcomes is still largely unknown. Nevertheless, the use of chemotherapy during pregnancy has been associated with placenta-related adverse outcomes. In particular, an increased risk of fetal growth restriction (FGR) has been associated with prenatal chemotherapy exposure, but this may partially be explained by other disease-related factors, such as rapid tumor growth (Toledo et al. 2006) or possibly maternal malnutrition and immunosuppression. Hence, a strength of the current study is that it provides mechanistic information on placental toxicity of chemotherapy without disease-associated confounding factors. Van Calsteren and colleagues looked into the possible pathogenesis of FGR after chemotherapy exposure, among which were both classical cytostatics and targeted therapies. They found that in FGR cases, chemotherapy exposure both occurred earlier in pregnancy and lasted longer. In chemotherapy-exposed placentas (both with and without FGR), they found an increase in oxidative DNA damage in trophoblasts, which seemed to be higher than in unexposed FGR placentas (Verheecke et al. 2018). Additionally, Abbelar et al. described histopathological findings in 12 placentas with chemotherapy exposure in the 2nd and 3rd trimesters that were indicative of placental underdevelopment (Abellar et al. 2009). In both studies, the number of cases was low and large heterogeneity existed in chemotherapeutic agents and treatment duration. Histopathological studies with a higher number of chemotherapy-exposed placentas are necessary to overcome the current gap in the in vitro–in vivo extrapolation of placental toxicity and pregnancy outcome.

Investigating the effect of drugs on endocrine function in placental trophoblast is of major importance, since placental endocrine function is critical for pregnancy maintenance. Progesterone is crucial for endometrium receptivity and immunotolerance, while estrogens regulate angiogenesis and maternal metabolic changes (Costa 2016). Additionally, abnormal steroidogenesis has been associated with placenta-related adverse pregnancy outcomes, such as preeclampsia (Berkane et al. 2018). Here, we studied inhibition of steroidogenesis in syncytialised BeWo cells successfully and found that the increase in progesterone release after forskolin treatment is in line with previous studies (Drwal et al. 2018). From a methodological point of view, we added to this that the amount of DMSO interferes with forskolin-induced progesterone release. However, small amounts of DMSO cannot be avoided when performing experiments using forskolin. Of the studied anticancer drugs, sunitinib (1 µM and 3 µM) was able to decrease progesterone levels to some extent, which preceded loss of cell viability.

We were able to detect estrogens in the culture medium of the cell lines studied, but could not quantify the estrogen concentrations due to the low quantities present. Although the placenta seems to be capable of de novo estrogen synthesis from cholesterol present in FCS (Escobar et al. 2011), estrogen production is generally thought to be largely dependent on fetal androgens. Addition of precursor androgens achieved by co-culturing adrenal cells with syncytialised BeWo cells was shown to increase estrogen synthesis substantially (Drwal et al. 2018). In term placental explants, a decrease was found of progesterone release of approximately 50-fold over the first four days of culture (Sato et al. 2015). These authors also observed a decrease in CYP19A1 expression levels in explants compared to freshly isolated tissue, consistent with a drop in estrone and estradiol levels. In the current study, we confirmed a decrease in progesterone and estrone release over seven days of culture. Effects on progesterone release independent of tissue viability were not observed, and the large variation in the data makes it difficult to draw firm conclusions. Interestingly, estrone release remained stable even when tissue viability was fully reduced, suggesting that the continued release takes place from villous tissue from extracellular reservoirs. Steroids can bind to Sex Hormone-Binding Globuline (SHBG) (Hammond 2016), and SHBG can associate with fibulin, an extracellular matrix component (Ng et al. 2006). Release of estrone from fibulin-associated SHBG may represent such a reservoir in our experiments. This phenomenon could also explain the minimal estradiol levels in our explant cultures as the binding affinity to SHBG, and consequently its release from tissue, is lower compared to estrone. Future studies should address the optimization of culture conditions to preserve enzyme activity in placental explants. Placental explants are highly sensitive to oxygen levels (Miller et al. 2005; Treissman et al. 2020), which are known to influence aromatase expression (Jiang et al. 2000). Also, the addition of forskolin may enhance CYP19A1 activity as we observed a forskolin-induced increase in estrone release (Fig. 3). Again, supplementation with androgens may also be necessary. Taken together, syncytialised BeWo cells appear to be the better model to study endocrine toxicity effects over the use of explants.

In conclusion, most anticancer drugs affected the viability of placental explants and trophoblast cell lines at clinically relevant concentrations. For paclitaxel and doxorubicin, however, we observed large differences between the models. The placental explant model needs to be further improved for studying effects on steroidogenesis, whereas syncytialised BeWo cells proved to be suitable for this purpose. Sunitinib was the only drug that reduced progesterone release. Given the observed effects on cell and tissue viability, the placenta should be recognized as a potential target organ for toxicity of anticancer drugs.

## Electronic supplementary material

Below is the link to the electronic supplementary material.

Supplementary file1 (DOCX 385 kb)

## Data Availability

The datasets of this study are available from the corresponding author upon reasonable request.

## Code availability

Not applicable for this study.
