# Supplementary material for: Toxicity of anticancer drugs in human placental tissue explants and trophoblast cell lines
Source: Arch Toxicol. 2020 Oct 20;95(2):557–71. doi: 10.1007/s00204-020-02925-w (PMC7870638; doi:10.1007/s00204-020-02925-w)
Supplement: Supplementary file 1 — Supplementary file1 (DOCX 385 kb) [file 204_2020_2925_MOESM1_ESM.docx]

**Fig. S1 DMSO does not affect viability and steroid release rate in villous explants**

MTT absorbance after 72h in control conditions without DMSO does not deviate from baseline MTT absorbance at 0h (a). Progesterone (b) and Estrone (c) relative release per well per 24h as compared to baseline conditions (0h) may be altered by DMSO after 48h and 72h exposure. All bars represent mean ± SEM of three individual experiments performed in triplicate wells. Statistical analysis was performed using a two-way repeated measures ANOVA and the effects of DMSO were compared to control per time point using a Bonferroni post-hoc test. *P<0.05 (c, d)

**Fig. S2 The effects of tyrosine kinase inhibitors and cytostatic drugs on progesterone release in villous explants over time**

The effect of tyrosine kinase inhibitors (a-d) and cytostatics (e-h) on progesterone release over time. Relative progesterone release rate per well per 24h as compared to baseline conditions (0h) was calculated and normalized against the corresponding control. Bars represent mean + SEM of three independent experiments performed in triplicate wells. Statistical analysis was performed using a two-way repeated measures ANOVA and the Bonferroni post-hoc test was applied to compare treatment conditions with controls at each time point. *P<0.05 **P<0.01 ***P<0.001

**Fig. S3 The effects of tyrosine kinase inhibitors and cytostatic drugs on estrone release in villous explants over time**

The effect of tyrosine kinase inhibitors (a-d) and cytostatics (e-h) on estrone release over time. Relative estrone release rate per well per 24h as compared to baseline conditions (0h) was calculated and normalized against the corresponding control. Bars represent mean + SEM of three independent experiments performed in triplicate wells. Statistical analysis was performed using a two-way repeated measures ANOVA and the Bonferroni post-hoc test was applied to compare treatment conditions with controls at each time point. ***P<0.001

**Fig. S4 The addition of 0.1% DMSO does not alter the effects of cisplatin and carboplatin on cell viability in syncytialised BeWo cells**

The addition of 0.1% DMSO does not largely affect the effect of cisplatin (a) and carboplatin (b) on cell viability as compared to BeWo cells without DMSO in the culture medium after 48h exposure. Bars represent the mean + SEM of three independent experiments ach performed in triplicate wells. A regular two-way ANOVA was performed to assess the effect of the presence of DMSO on the inhibitory effects of cisplatin and carboplatin on cell viability. The Bonferroni post-hoc test was applied to compare treatment conditions with controls. **P<0.01 ***P<0.001
